# Acute worsening of glycemic control in a patient with type 2 diabetes and non‐small cell lung cancer after administration of lorlatinib

**DOI:** 10.1002/ccr3.5283

**Published:** 2022-01-20

**Authors:** Yoshio Nakano, Mai Miyasato‐Isoda, Iwao Gohma, Tomomi Fujisawa

**Affiliations:** ^1^ Department of Respiratory Medicine Sakai City Medical Center Sakai Japan; ^2^ Department of Diabetes Metabolism and Endocrinology Sakai City Medical Center Sakai Japan

**Keywords:** adverse event, hyperglycemia, lorlatinib, non‐small cell lung cancer, type 2 diabetes

## Abstract

A patient with non‐small cell lung cancer (NSCLC) exhibited extreme hyperglycemia after lorlatinib treatment. The present case highlights the importance of glucose monitoring during lorlatinib administration and intensifying hyperglycemia treatment.

## Graphical abstract

The present case indicated that 1) glucose monitoring is warranted during lorlatinib treatment and 2) the importance of intensifying hyperglycemia treatment after lorlatinib administration.

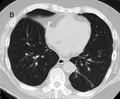

## 1 INTRODUCTION

The worldwide prevalence of diabetes mellitus and lung cancer is increasing. Lung cancer is a leading cause of death worldwide,^1^ and 8%–18% of cancer patients have been reported to have diabetes mellitus.^2^ Therefore, adequate management of comorbid diabetes is essential for the treatment of cancer. Approximately 5% of non‐small cell lung cancers (NSCLC) exhibit an aberrant gene arrangement for anaplastic lymphoma kinase (ALK).^3^, ^4^, ^5^ Lorlatinib, a third‐generation ALK tyrosine kinase inhibitor (TKI) that inhibits ALK and c‐ros oncogene 1 (*ROS1*), has been clinically available for patients with mutations/arrangements of the ALK gene, usually called ALK‐positive NSCLC.

The clinical use of lorlatinib is associated with relatively unique metabolic phenotype adverse events such as hypercholesterolemia (82.4%) and hypertriglyceridemia (60.7%).^6^ However, in a phase 2 study, hyperglycemia was observed as an adverse event in six (2.2%) out of 275 patients receiving lorlatinib,^7^ four in Grade 1–2 (1.5%), and two in Grade 3 (0.7%), but none in Grade 4 (based on the Common Terminology Criteria for Adverse Events CTCAE v.4.03). Herein, we report a case of NSCLC in a patient with well‐controlled diabetes mellitus whose glycemic control markedly deteriorated after the initiation of lorlatinib treatment, resulting in hyperglycemia equivalent to G4. However, lorlatinib treatment was continued without dose reduction by applying appropriate therapy for hyperglycemia.

## 2 CASE REPORT

A 70‐year‐old man with type 2 diabetes mellitus, treated with vildagliptin 50 mg, a dipeptidyl peptidase‐4 inhibitor (DPP4I) with an HbA1c level of 5.4%–6.7%, was diagnosed with abnormal lung opacity by computed tomography (CT) when examined for cough in January X‐2 year. He was subsequently diagnosed with cT4N3M1a stage IVA lung adenocarcinoma (NSCLC). The patient was positive for ALK gene translocation, and alectinib was started as the first‐line therapy, with a response of CR (graded New Response Evaluation Criteria in Solid Tumors: revised RECIST guideline [version 1.1]) without significant adverse events. Chest CT in March X year showed an increase in the size of the lower left lobe tumor that was judged as progressive disease (PD). Lorlatinib 100 mg/day second‐line therapy was administered in March X year. His blood glucose control was stable, with a random blood glucose level of 167 mg/dl and an HbA1c level of 6.4%. At the follow‐up visit on day 92 after initiation of lorlatinib, he had thirst without any other symptoms; his glycemic control had markedly deteriorated: 545 mg/dl of random blood glucose and HbA1c 16.1%. He was admitted to the hospital for treatment of hyperglycemia (Table 1). The patient's characteristics and symptoms on admission included height 171.5 cm, weight 83.5 kg (82.2 kg before the start of lorlatinib), body mass index 28.4, clear consciousness, blood pressure 165/88 mmHg, heart rate 104/min, respiratory rate 16 breaths/min, SpO_2_ 97% (room air), and body temperature 35.9°C.

**TABLE 1:** Changes in metabolic parameters: Blood glucose, HbA1c, TG, LDL, and Bodyweight

year/month	X−1/6~X/2	X/3	X/4	X/5	X/6	X/7	X/8	X/9
BG (mg/dl)	120–160	170	155	482	545	87	121	87
HbA1c (%)	5.8–6.7	6.4			16.1	12.5	8.9	7.0
TG (mg/dl)		451	451	528	712	262	277	342
LDL (mg/dl)	67	63	194	239	252	180	151	205
BW (kg)		82.2			83.5			

Abbreviations: BG, blood glucose; BW, body weight; LDL, low‐density lipoprotein cholesterol; TG, triglyceride.

**FIGURE 1:**
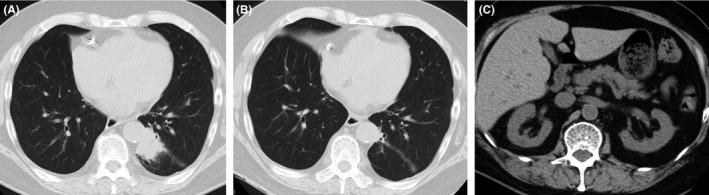
Chest and abdominal computer tomography (CT). (A) Chest CT in March X years showed an increase in tumor size in the left lower lobe. (B) Chest CT on June X showed a reduction in tumor size. (C) No pancreatic tumor showed on abdominal CT in June X

Blood analysis results at admission were as follows: leukocyte count, 7540/μL; Hb, 10.3 mg/dl; Cr, 1.26 mg/dl; CEA, 13.0 Â ng/ml; CYFRA, 4.3 ng/ml; anti‐GAD antibody, below the measurement sensitivity; and C‐peptide 7.3 ng/ml. Chest CT showed a reduction in tumor size (Figure 1).

Post‐hospital course: At admission, his blood glucose level was as high as 500 mg/dl. The patient did not exhibit obvious overeating or weight gain, and the anti‐GAD antibody test results were negative. CT imaging demonstrated no signs of pancreatic tumor (Figure 1) and the serum C‐peptide level was maintained; therefore, we suspected that the type 2 diabetes mellitus was exacerbated by the oral administration of lorlatinib (Table 1). The Naranjo^8^ score was 5 (Table 2), suggesting that lorlatinib was likely the cause of this adverse event.

**TABLE 2:** Naranjo Adverse Drug Reaction Probability Scale

Question	Yes	No	Do Not Know	Score
1. Are there previous conclusive reports on this reaction?	+1	0	0	0
2. Did the adverse event appear after the suspected drug was administered?	+2	−1	0	+2
3. Did the adverse reaction improve when the drug was discontinued or a specific antagonist was administered?	+1	0	0	0
4. Did the adverse event reappear when the drug was re‐administered?	+2	−1	0	0
5. Are there alternative causes (other than the drug) that could on their own have caused the reaction?	−1	+2	0	+2
6. Did the reaction reappear when a placebo was given?	+1	+1	0	0
7. Was the drug detected in blood (or other fluids) in concentrations known to be toxic?	+1	0	0	0
8. Was the reaction more severe when the dose was increased or less severe when the dose was decreased?	+1	0	0	0
9. Did the patient have a similar reaction to the same or similar drugs in any previous exposure?	+1	0	0	0
10. Was the adverse event confirmed by any objective evidence?	+1	0	0	+1
Total Score:				5

Note

Adverse drug reaction probability is as follows: Certain>9; Probable 5–8; Possible 1–4; Unlikely 0.

To control his hyperglycemia, intensive insulin therapy (multiple daily injections of insulin) was initiated while lorlatinib treatment was continued. Based on his reduced renal function, vildagliptin was changed to linagliptin. Finally, his blood glucose level was controlled by injecting a rapid‐acting insulin analog (Insulin Lispro 30–20–20 units) and basal insulin (glargine 18 units) at bedtime, with oral administration of linagliptin after breakfast. His fasting blood glucose level was approximately 160 mg/dl, and the level before the meal was 170–200 mg/dl. To treat hypertriglyceridemia and hyperlipidemia, pemafibrate and ezetimibe were started in addition to pitavastatin. He was discharged from the hospital on the 12th day. Subsequently, partial response (PR) to lorlatinib was maintained for 6 months, and his glucose levels and lipid profiles were well controlled, with HbA1c levels of 7.0%–6.6%, TG levels of 342–251 mg/dl, and LDL levels of 304–151 mg/dl.

## 3 DISCUSSION

The present case exhibited extreme hyperglycemia after administration of lorlatinib, a new anti‐cancer medication class for NSCLC. Despite its effects on body weight, lorlatinib causes well‐known adverse effects on lipid metabolism but less frequent and less severe effects on glucose metabolism. The effectiveness of lorlatinib on lung cancer in the present case was apparent, and the patient continued lorlatinib treatment with an adequate intensification of the diabetes treatment. The present case highlights two clinical issues related to lorlatinib therapy in patients with diabetes mellitus.

First, the administration of lorlatinib could lead to the deterioration of glycemic control. Excessive hyperglycemia may be more clinically relevant than deterioration of dyslipidemia. The former could lead to hyperglycemia‐associated symptoms and hypovolemia over a shorter period than dyslipidemia and may affect the quality of life (QOL) as it did in the present case. Interestingly, one patient was reported to have aggravated glycemic control after administration of ceritinib, another ALK inhibitor, for lung cancer treatment.^9^ Taken together, attention needs to be paid to glucose metabolism, in addition to the lipid profile, in patients taking lorlatinib; monitoring of glucose levels is warranted for patients receiving this medication, especially for those with diabetes mellitus.

Another clinical issue is the importance of intensifying diabetes treatment at elevated glucose levels after lorlatinib treatment. In the present case, the effect of lorlatinib on NSCLC was clinically evident for 3 months, at which time he exhibited excessive hyperglycemia equivalent to Grade 4. However, lorlatinib treatment was continued together with an adequate intensification of the therapy for hyperglycemia. Intensive insulin treatment was started with a relatively high dose of insulin (88 units/day: 1.05 units/kg/day), in addition to DPP4I. He continued with lorlatinib for 6 months following the discharge and his lung cancer had maintained PR within 6 months. Thus, adequate management of diabetes allowed the continuation of lorlatinib therapy, after the diabetes treatment was intensified due to elevated glucose levels during the administration of lorlatinib.

It is of clinical concern that lorlatinib could be associated with the deterioration of glucose metabolism. In the phase 2 study, hyperglycemia was reported in only 6 of the 275 patients; four patients (1.5%) in G1–2, two patients (0.7%) in G3, and none (0%) in G4.^6^ In contrast, an increase in body weight was more frequently detected in the phase1/2 study, with 87 individuals (30.9%) among the 282 patients having a 10%–20% increase in their baseline body weight, and 38 (13.5%) had a >20% increase in their baseline body weight.^7^ In the present case, the observed body weight gain during the detection of his extreme hyperglycemia was merely 1 kg, compared to before starting lorlatinib. However, his body weight increase (1 kg) was probably counter‐balanced by his excessive hyperglycemia leading to bodyweight reduction. Given that he had no sign of edema, this incremental increase in body weight was likely due to increased adipose tissue that could enhance insulin resistance, leading to hyperglycemia. The worsening of his insulin resistance is evidenced by the need for a relatively high dose (88 units/day: 1.05 units/kg/day) insulin injection. Although our search did not find any case reports of exacerbation of hyperglycemia related to lorlatinib, a relatively common frequency of body weight increase (44.4%) indicates that more cases of deteriorated glucose control after lorlatinib administration probably occurred. Diabetes is a chronic disease, and glycemic control can change due to various factors. Although there were no side effects such as psychiatric symptoms or changes in lifestyle due to lorlatinib, only glycemic control deteriorated, so it was considered to be a side effect due to lorlatinib.

In summary, the present case exhibited extreme hyperglycemia after lorlatinib treatment for ALK‐positive NSCLC. By intensifying his diabetes treatment, he was able to continue taking lorlatinib with PR after discharge. The present case showed two relevant points: glucose monitoring is warranted after the start of treatment with lorlatinib, especially for those with diabetes mellitus, and adequate management of diabetes can allow the continuation of lorlatinib for cases in which lung cancer is effectively treated. The mechanism by which lorlatinib causes hyperglycemia remains unclear and should be examined in the future.

## CONFLICT OF INTEREST

The authors state that they have no conflict of interest.

## AUTHOR CONTRIBUTIONS

The first two authors, YN and MMI, were involved in the case of the patient in the ward, under the supervision of IG and TF. All authors contributed to the final manuscript regarding the interpretation of the outcomes and conclusions. YN searched PubMed for relevant studies and wrote the manuscript under the supervision of MMI, IG, and TF.

## CONSENT

Written informed consent was obtained from the patient for the publication of this case report and accompanying images.

## Data Availability

Data sharing is not applicable to this article as no new data were created or analyzed in this study.
